# Chronic temperature stress inhibits reproduction and disrupts endocytosis via chaperone titration in *Caenorhabditis elegans*

**DOI:** 10.1186/s12915-021-01008-1

**Published:** 2021-04-15

**Authors:** Rosemary N. Plagens, Isiah Mossiah, Karen S. Kim Guisbert, Eric Guisbert

**Affiliations:** Department of Biomedical and Chemical Engineering and Sciences, Florida Institute of Technology, Melbourne, FL USA

**Keywords:** Heat shock response, Chronic stress, Endocytosis, Trafficking, Chaperones

## Abstract

**Background:**

Temperature influences biology at all levels, from altering rates of biochemical reactions to determining sustainability of entire ecosystems. Although extended exposure to elevated temperatures influences organismal phenotypes important for human health, agriculture, and ecology, the molecular mechanisms that drive these responses remain largely unexplored. Prolonged, mild temperature stress (48 h at 28 °C) has been shown to inhibit reproduction in *Caenorhabditis elegans* without significantly impacting motility or viability.

**Results:**

Analysis of molecular responses to chronic stress using RNA-seq uncovers dramatic effects on the transcriptome that are fundamentally distinct from the well-characterized, acute heat shock response (HSR). While a large portion of the genome is differentially expressed ≥ 4-fold after 48 h at 28 °C, the only major class of oogenesis-associated genes affected is the vitellogenin gene family that encodes for yolk proteins (YPs). Whereas YP mRNAs decrease, the proteins accumulate and mislocalize in the pseudocoelomic space as early as 6 h, well before reproduction declines. A trafficking defect in a second, unrelated fluorescent reporter and a decrease in pre-synaptic neuronal signaling indicate that the YP mislocalization is caused by a generalized defect in endocytosis. Molecular chaperones are involved in both endocytosis and refolding damaged proteins. Decreasing levels of the major HSP70 chaperone, HSP-1, causes similar YP trafficking defects in the absence of stress. Conversely, increasing chaperone levels through overexpression of the transcription factor HSF-1 rescues YP trafficking and restores neuronal signaling.

**Conclusions:**

These data implicate chaperone titration during chronic stress as a molecular mechanism contributing to endocytic defects that influence multiple aspects of organismal physiology. Notably, HSF-1 overexpression improves recovery of viable offspring after exposure to stress. These findings provide important molecular insights into understanding organismal responses to temperature stress as well as phenotypes associated with chronic protein misfolding.

**Supplementary Information:**

The online version contains supplementary material available at 10.1186/s12915-021-01008-1.

## Background

The fate of an organism can rest on its ability to accurately sense and respond to stress. A ubiquitous stress that often disrupts homeostasis is temperature fluctuation [1]. Consequently, biological systems have a variety of mechanisms to re-establish homeostasis when faced with elevated temperatures. These adaptations to temperature can be both evolutionary and organismal. Evolutionary adaptations are driven by selection of heritable genetic changes. Organismal responses are driven by cellular and molecular mechanisms.

At the organismal level, animals exhibit changes in behavior, autonomic reflexes (e.g., sweating), cardiovascular function, and neuroendocrine signaling when exposed to elevated temperatures [2]. For livestock, temperature is one of the largest stressors in animal production, and organismal response mechanisms are typically detrimental to performance due to their high metabolic costs [3, 4]. Heat stress decreases growth, reproduction, milk production, and meat production, while increasing the occurrence of disease. Furthermore, organisms have evolved to survive specific ranges of temperatures, and an increase of only a few degrees is predicted to result in the extinction of as many as 16% of all species [5].

At the molecular level, elevated temperatures disrupt protein-folding homeostasis, or proteostasis [6]. Proteostasis is normally maintained by an extensive network of factors collectively known as the proteostasis network, which includes pathways that regulate the synthesis, folding, trafficking, and degradation of proteins. However, many proteins adopt native states that are only marginally stabilized, such that an increase of only 4 °C destabilizes the average protein by ~ 20% in *Escherichia coli* [7]. To deal with the massive accumulation of misfolded proteins during temperature stress, all organisms utilize an adaptative response known as the heat shock response (HSR) [8]. This highly conserved pathway is mediated by the transcription factor heat shock factor 1 (HSF1). Mutations in HSF1 cause thermosensitivity, whereas overexpression of HSF1 enhances thermotolerance [9, 10].

An important class of HSF1-regulated genes is the molecular chaperone family that assists in protein folding [11, 12]. In the absence of stress, chaperones participate in de novo folding of newly synthesized proteins and in cellular processes that require assembly and disassembly of macromolecular complexes. For example, chaperones are required for several steps of endocytosis [13–15]. The initial invaginations of coated pits, dissociation of clathrin during vesicle uncoating, and the stabilization of clathrin after dissociation are regulated by the chaperone HSC70. Another chaperone, RME-8, is important for downstream steps of clathrin-mediated endocytosis and is a shared regulator of both receptor-mediated endocytosis (RME) in oocytes and fluid-phase endocytosis in coelomocytes in *Caenorhabditis elegans* [16–19]. In the presence of stress, chaperones help to repair misfolded or damaged proteins [20]. This serves to titrate specific chaperones away from an inhibitory complex with HSF1. Therefore, the activity of HSF1 is intimately linked to the protein-folding state of the cell. Conditions other than temperature stress similarly lead to chaperone titration. During aging, a substantial decline in the capacity of the proteostasis network is attributed to age-associated accumulation of misfolded proteins [21]. Numerous neurodegenerative diseases are also associated with extended chaperone titration [22].

The consequences of stress are fundamentally distinct depending on the degree and duration of the stress. The HSR has been extensively studied at the molecular level using a variety of model systems exposed to acute, severe temperature stress. However, physiological responses to temperature stress in metazoans are typically observed during mild stress on much longer timescales. The connection between the molecular events that take place during acute stress and the organismal-level responses to chronic stress remains to be fully characterized. Recently, correlations have been made between molecular and organismal responses to temperature stress. For example, we have shown that a chronic, 4-week exposure to thermal stress in *Hippocampus erectus* seahorses results in altered feeding frequency and overall ventilation rates, along with increased expression of HSR genes and genes involved in regulating reproduction [23]. Additionally, the advent of next-generation sequencing and genomic information has enabled a multitude of studies to examine transcriptomic responses to temperature stress in a variety of metazoans [24–26]. While these studies generate a treasure trove of data, this methodology is limited to providing descriptive correlations.

The multicellular, transparent nematode *C. elegans* is an ideal candidate for examining the effects of temperature stress at the molecular, cellular, tissue-specific, and organismal levels. An acute, nearly lethal temperature stress (1 h at 33–35 °C) that elicits the HSR has no reported ill effects on organismal fecundity in *C. elegans* [27–29]. In contrast, a chronic, mild temperature stress (48 h at 28 °C—a few degrees above the normal growth temperatures of 15–25 °C) completely inhibits reproduction while only mildly affecting phenotypes such as viability and motility [30, 31]. Here, we combine genetics and transcriptomic analyses with cellular and organismal phenotypes to identify molecular mechanisms driving the dramatic differences in fate between acute and chronic stress.

## Results

### Chronic heat stress inhibits egg laying and elicits distinct transcriptomic responses

**Fig. 1:**
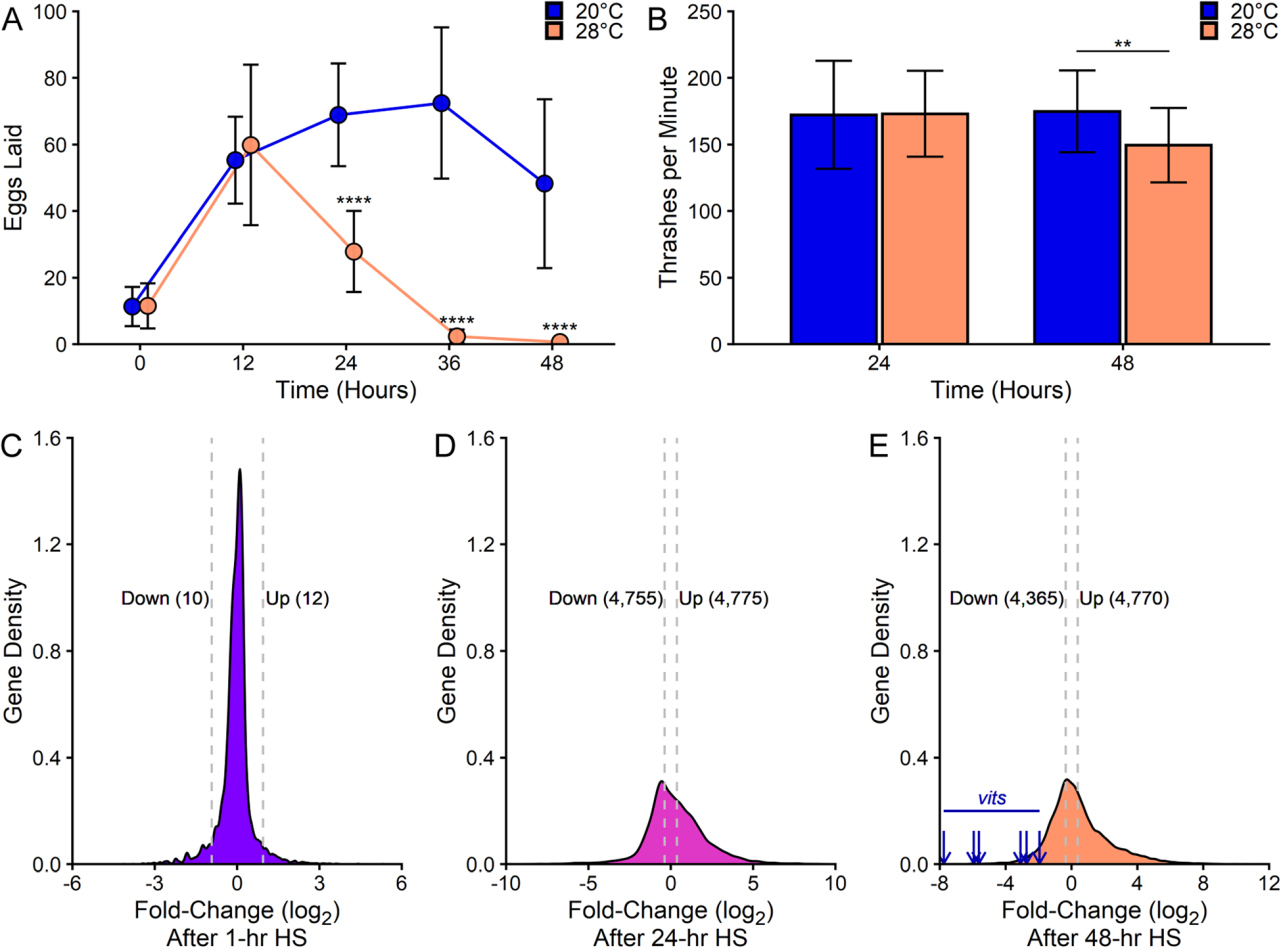
Chronic heat stress inhibits reproduction and affects global gene expression. Chronic exposure of day 1 adult N2 wild-type (WT) worms to 28 °C heat stress (HS) inhibits the number of eggs laid (**a**), mildly affects motility (**b**), and alters global gene expression (**c**–**e**) compared to 20 °C control worms. Data in **a** and **b** represent mean ± SD of *n* = 30 worms, collected across three independent trials. ** *p* < 0.01, **** *p* < 0.0001 (Student’s *t* test versus 20 °C at the same timepoint). Data in **c**–**e** represent densities of genes at each fold-change (log_2_) value relative to the 0-h (20 °C) controls following 1-h (**c**), 24-h (**d**), or 48-h (**e**) 28 °C HS as determined by RNA-seq analysis. Dashed vertical lines denote upper and lower expression limits of the genes that were up- or down-regulated, with the total number of differentially expressed genes in each category in parentheses. Arrows in **e** represent the fold-change expression of the six *vit* genes

Previous studies have shown that worms display a reproductive defect after prolonged exposure to mild heat stress (HS) [30, 31]. The temperature shifts in those studies were initiated prior to the onset of egg laying. However, more recent work showed that multiple stress responses attenuate at the onset of egg laying, such that stress-inducible gene expression in reproductive adults is half that of pre-reproductive worms [32]. Therefore, we first established organismal responses to chronic temperature stress after this final stage of development. Egg-laying adults were evaluated for reproduction and motility over a 2-day HS time course that reflects ~ 10% of the *C. elegans* lifespan. Worms exposed to 28 °C HS laid similar numbers of eggs as the control worms (20 °C) during the first 12 h, but only ~ 40% as many eggs between the 12- and 24-h HS timepoints (Fig. 1a). After the 24-h timepoint, the HS worms had essentially ceased reproduction. Despite the shutdown of reproduction, worms displayed no other obvious defects. This suggests that reproduction was not inhibited as a result of a catastrophic decline in worm health. To quantitatively assess whether chronic HS impacted motility, we used a thrashing assay. Exposure to 28 °C HS did not affect motility after 24 h and only had a slight effect (< 15%) after 48 h (Fig. 1b). Therefore, a dramatic loss of reproductive capacity but not motility is evident during chronic temperature stress after the onset of egg laying.

To identify molecular changes in these chronic stress conditions, the transcriptome was characterized using RNA-sequencing from three biological replicates at four timepoints: 0 h (20 °C control), 1 h, 24 h, and 48 h at 28 °C HS. These timepoints were selected to compare the effects of acute (1-h) and chronic (24- and 48-h) exposures to 28 °C HS, based on the distinct phenotypic changes observed in reproduction and motility. Each sample generated between 39 and 59 million clean reads, with 5.91 to 8.98 G of clean bases. The samples each had a Q20 score > 97.4% and the Pearson correlation coefficients between the biological replicates ranged between 0.973 and 0.994, indicating high sequencing quality and reproducibility. Differentially expressed genes were identified using the DESeq R package comparing each HS timepoint to the 0-h 20 °C controls (see Additional file 1) [33]. Genes with an adjusted (Benjamini-Hochberg) *p* value < 0.05 were considered differentially expressed [34].

Analysis of the RNA-seq data revealed that 1 h at 28 °C caused differential expression of only a few genes (10 genes down-regulated; 12 genes up-regulated) (Fig. 1c). The down-regulated genes are largely uncharacterized, but include three genes with predicted solute carrier activity, two with predicted roles in coenzyme A processes, and several with predicted catalytic activity. The up-regulated genes include six heat shock proteins (HSPs) as expected, as well as four genes of unknown function and two genes with diverse roles (*ckb-2* and *cebp-1*). This dataset, while using a duration commonly used for acute HS (1 h), is distinct from standard acute HS protocols that employ higher temperatures and observe larger changes in gene expression. In contrast, 24 h of 28 °C HS led to down-regulation of 4755 genes and up-regulation of 4775 genes (Fig. 1d). Approximately 28% of the differentially expressed genes (697 genes down; 1934 genes up) experienced a ≥ 4-fold change in expression. The 48-h 28 °C HS led to down-regulation of 4365 genes and up-regulation of 4770 genes (Fig. 1e), ~ 33% of which (749 genes down; 2303 genes up) were altered by ≥4-fold. Venn diagrams showed that all of the genes differentially expressed during 1-h 28 °C HS were shared with at least one of the longer HS conditions, whereas 30% of the 24-h and 27% of the 48-h 28 °C HS genes were uniquely regulated by those timepoints (see Additional file 2: Fig. S1 and Additional file 3). Together, these data reveal extensive, duration-specific remodeling of the transcriptome in response to chronic stress. Furthermore, the molecular responses to chronic, mild temperature stress extend beyond the well-characterized heat shock response (HSR). Only 8% of the genes regulated during 48-h 28 °C HS were shared with a previously published gene set representing a classical acute HSR [35] (see Additional file 2: Fig. S2 and Additional file 3).

To determine which cellular pathways were affected during chronic HS, gene ontology (GO) enrichment analysis was used (see Additional file 2: Fig. S3 and Additional file 3) [36, 37]. The 24-h HS gene enrichments showed an up-regulation in stress responses, such as defense and immune system responses, along with down-regulation of metabolic processes and nucleotide binding. Interestingly, both the 24-h and the 48-h HS timepoints elicited down-regulation of genes associated with the structural constituent of the cuticle, with the 48-h HS also up-regulating many cuticle-associated genes, suggesting a cuticle restructuring event in response to chronic HS.

The most dramatic phenotypic effect of chronic stress is inhibition of egg laying. If the reproductive cessation during chronic HS was being driven by transcriptional shutdown of oogenesis, we would expect to see a general repression of these genes. However, GO analyses did not reveal enrichment for reproduction-associated processes. To investigate this further, the genes involved in reproduction were specifically queried by examining the expression levels of oogenesis-enriched genes, as described by Reinke et al. [38]. Together, the 920 oogenesis-enriched genes identified in our RNA-seq data showed no change in expression at the 1-h timepoint, slight repression at 24 h, and a smaller degree of repression at the 48-h HS timepoint (see Additional file 2: Figs. S4A-C). Among these genes, ~ 3.8% were affected at least 4-fold by exposure to 28 °C HS for 24 or 48 h. Therefore, our data indicate that the cessation of reproduction during chronic HS does not involve an overall shutdown of oogenesis-enriched gene expression.

### Chronic heat stress disrupts endocytosis of yolk by oocytes

**Fig. 2:**
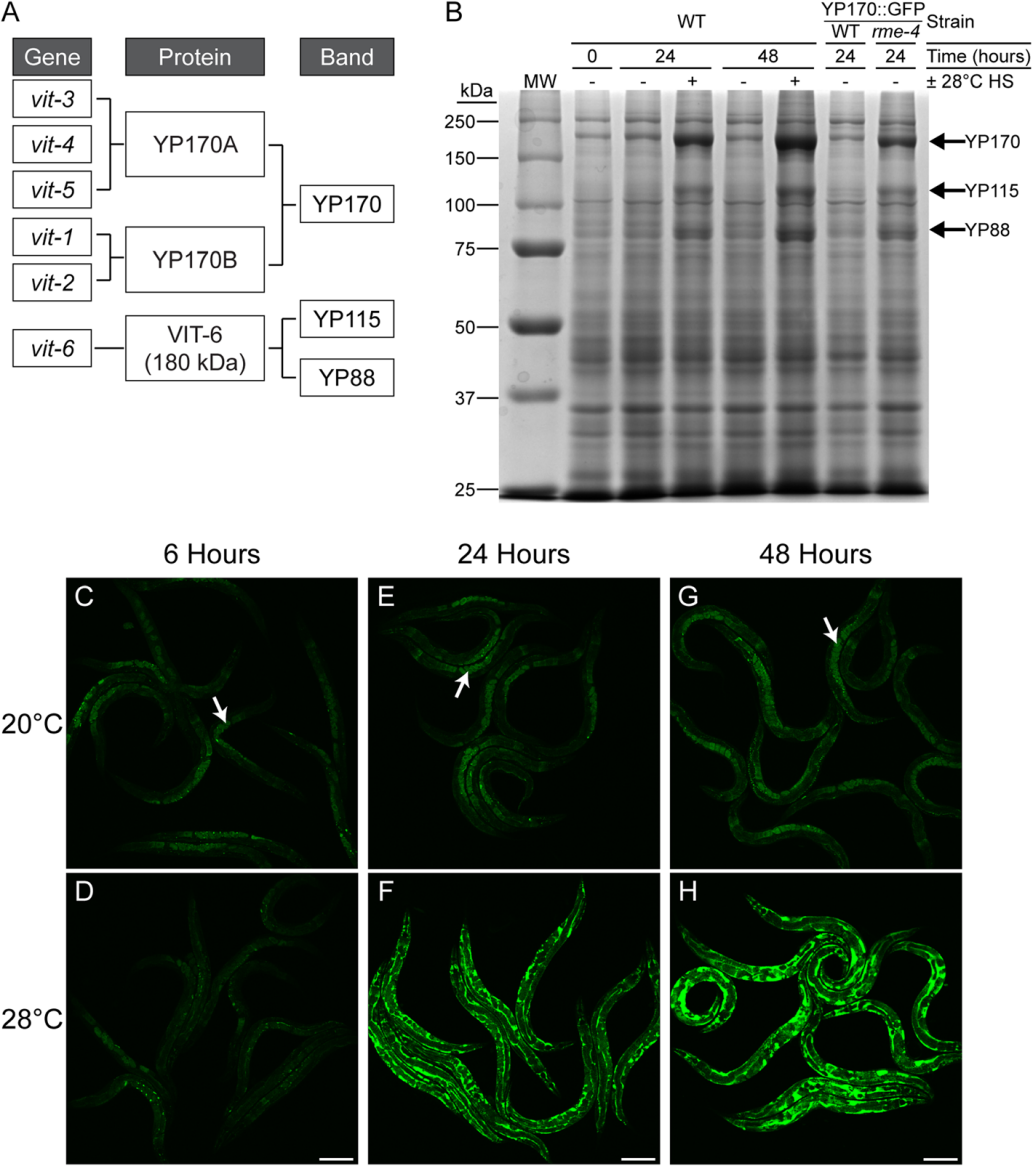
Chronic heat stress causes yolk protein accumulation and mislocalization. **a** A schematic showing the relationship between the six *vit* genes, their initial protein products, and the three major yolk protein (YP) bands. **b** Coomassie-stained SDS-PAGE reveals accumulation of all three major YP species following chronic exposure to 28 °C HS. Arrows indicate YP170, YP115, and YP88 bands. The *rme-4(b1001)* mutant YP170::GFP strain (RT362) was included for YP band identification. Shown is a representative image from three independent trials. MW: molecular weight marker; WT: wild-type. **c**–**h** Chronic exposure to 28 °C HS causes accumulation and mislocalization of the YP170::GFP (RT130) fluorescent reporter. Arrows in **c**, **e**, and **g** demonstrate proper localization of YP170::GFP in embryos at the control temperature. Shown are representative images from three independent trials with *n* ≥ 10 worms in each trial. Scale bar: 150 μm

Among the most strongly repressed genes at the 48-h HS timepoint was the six-member vitellogenin gene family (*vit-1* through *vit-6*) that encodes the yolk proteins (YPs) (Fig. 1e, arrows), with levels ranging from a ~ 3.9-fold to 216.8-fold decrease in expression compared to controls. The *vit* genes and their YP products play important roles in providing nutrients to developing oocytes [39–41]. In *C. elegans*, the six *vit* genes produce three major YP species—YP170, YP115, and YP88—that are named according to their molecular weights (see schematic in Fig. 2a). The YP170 species is composed of two protein products: YP170A, encoded by *vit-3*, *vit-4*, and *vit-5*; and YP170B, encoded by *vit-1* and *vit-2*. The other two protein species—YP115 and YP88—are cleaved in the pseudocoelom (body cavity) from a 180-kDa precursor protein produced by *vit-6* [42, 43].

Since *vit* expression was decreased during chronic HS, YP levels were analyzed by SDS-PAGE to determine whether they also decreased [44, 45]. In contrast to the repression of YP mRNA levels, chronic 24- and 48-h HS led to accumulation of all three major YP species when compared to age-matched controls (Fig. 2b). YP identities were validated by comparison with a mutant known to accumulate YPs (compare YP170::GFP;*rme-4* mutants to YP170::GFP;WT in Fig. 2b) [46].

The striking accumulation of YPs seen in adults could occur in one of the three locations involved in the YP trafficking pathway: the intestine (site of synthesis), the embryos (site of uptake), or the pseudocoelomic space that separates the intestine from the gonad [44, 47, 48]. To determine the location of YP accumulation, a fluorescent reporter containing YP170 fused with GFP (YP170::GFP) was used (Fig. 2c–h) [47]. Compared to 20 °C controls, where YP170::GFP was localized in the oocytes (see arrows in Fig. 2c, e, g), exposure to 28 °C HS for 24 h (Fig. 2f) or 48 h (Fig. 2h) resulted in increased levels of YP170::GFP in the pseudocoelom but not in the intestine or the oocytes (see Additional file 2: Fig. S5 for higher magnification at the 24-h timepoint). As oocytes normally take in yolk using receptor-mediated endocytosis, the pseudocoelomic accumulation of YPs during HS phenocopies mutations in endocytosis [47]. Importantly, this phenotype can be observed after a 6-h HS exposure, with prominent YP170::GFP puncta around the embryos and reduced YP170::GFP intensity inside the embryos compared to the 20 °C controls (Fig. 2c vs 2d). This finding revealed that YP accumulation preceded, and was therefore not a consequence of, the egg-laying defect that developed between 12 and 24 h of HS.

### Chronic heat stress disrupts endocytosis in coelomocytes and affects neuronal signaling

**Fig. 3:**
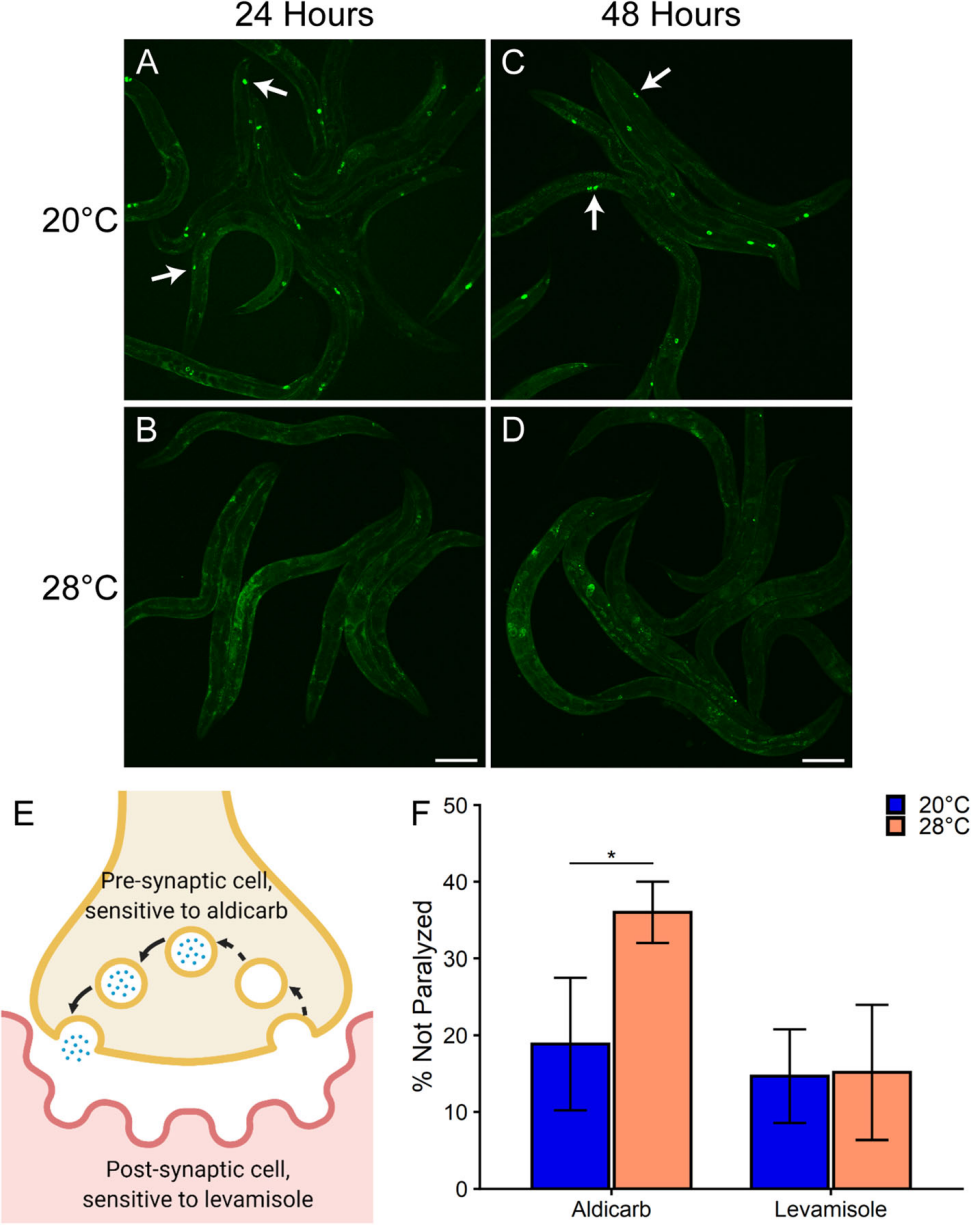
Chronic heat stress inhibits endocytosis in coelomocytes and disrupts neuronal signaling. **a**–**d** Chronic exposure to 28 °C HS (**b** and **d**) disrupts coelomocyte endocytosis compared to 20 °C controls (**a** and **c**) as observed in *myo-3*p::*ssGFP* (GS1912) adults. Shown are representative images from three independent trials with *n* ≥ 10 worms. Arrows in **a** and **c** demonstrate proper localization of ssGFP in coelomocytes at the control temperature. Scale bar: 150 μm. **e** A schematic showing the cycle of synaptic vesicle formation and neurotransmitter release at a neuromuscular junction. Synaptic vesicle components are normally recycled through endocytosis (dashed arrows). **f** Exposure of WT (N2) adults to 48-h 28 °C HS increases resistance to aldicarb, but not to levamisole, compared to 20 °C controls as measured using paralysis assays. Data represent the mean ± SD of *n* = 3 independent trials. * *p* < 0.05 (Student’s *t* test)

To determine whether disruption of YP trafficking is indicative of a generalized defect in trafficking, an independent protein trafficking reporter was used. This reporter contains a muscle-specific myosin promoter that drives a GFP transgene containing a signal sequence (*myo-3*p::*ssGFP*). The ssGFP is secreted from the muscles into the pseudocoelom and then taken up via fluid-phase endocytosis into the six macrophage-like coelomocytes, where its fluorescence is readily observable [19]. Whereas fluorescence was localized in the coelomocytes at the control temperature (Fig. 3a, c), exposure of this reporter to 24- or 48-h 28 °C HS resulted in diffuse fluorescence across the pseudocoelom (Fig. 3b, d). Thus, this protein trafficking reporter with a distinct endocytic process revealed a second trafficking defect in a different target tissue.

However, trafficking of the *myo-3*p::*ssGFP* reporter could be indirectly affected by the high levels of accumulated YP in the pseudocoelomic space during chronic stress. To test this, we knocked down YP in the *myo-3*p::*ssGFP* reporter strain using RNA interference (RNAi) against *ceh-60*, a regulator of vitellogenin expression [49, 50]. While *ceh-60* RNAi effectively knocked down YP in the YP170::GFP reporter, which showed little to no GFP fluorescence (see Additional file 2: Figs. S6E-H), it did not affect the coelomocyte trafficking defect in the *myo-3*p::ssGFP reporter during exposure to 24-h 28 °C HS (Additional file 2: Figs. S6A-D). These results indicate that yolk accumulation in the pseudocoelom does not inhibit coelomocyte uptake and that a general defect in endocytosis occurs during chronic stress.

If endocytosis is globally disrupted during chronic stress, then neuronal signaling, which is heavily dependent on endocytosis, would also be affected. Such a defect would localize to the pre-synaptic junctions that rely on clathrin-mediated endocytosis to recycle synaptic vesicle components [51–53] (see schematic in Fig. 3e). Disruption of pre-synaptic versus post-synaptic neuronal signaling can be distinguished by sensitivity to the paralysis-inducing drugs aldicarb, an acetylcholinesterase inhibitor that acts on pre-synaptic signaling, and levamisole, a nicotinic acetylcholine receptor agonist that acts on post-synaptic signaling [54, 55]. Exposure to 48-h 28 °C HS caused a dramatic reduction in sensitivity to aldicarb, with ~ 36% of the stressed worms showing aldicarb resistance (not paralyzed) compared to only ~ 19% of the control worms remaining motile (Fig. 3f). In contrast, no difference was observed in the sensitivity to levamisole between the unstressed controls and the heat-stressed worms. These data suggest a pre-synaptic signaling defect during chronic stress, which supports the hypothesis that chronic stress globally disrupts endocytosis.

### Heat shock factor 1 (HSF-1) overexpression rescues chronic stress-induced endocytic disruptions

Several steps in endocytosis are dependent on molecular chaperones for assembly and disassembly of macromolecular complexes [14, 15]. These molecular chaperones are also important for protein folding during temperature stress and are responsible for negative regulation of the HSR in the absence of stress [11]. Activation of the HSR in our transcriptomic data indicates that chaperones are titrated by misfolded proteins during chronic stress. Therefore, we hypothesized that titration of chaperones away from their constitutive roles in endocytosis could be the mechanism causing disruption of protein trafficking and subsequent organismal phenotypes during chronic stress.

**Fig. 4:**
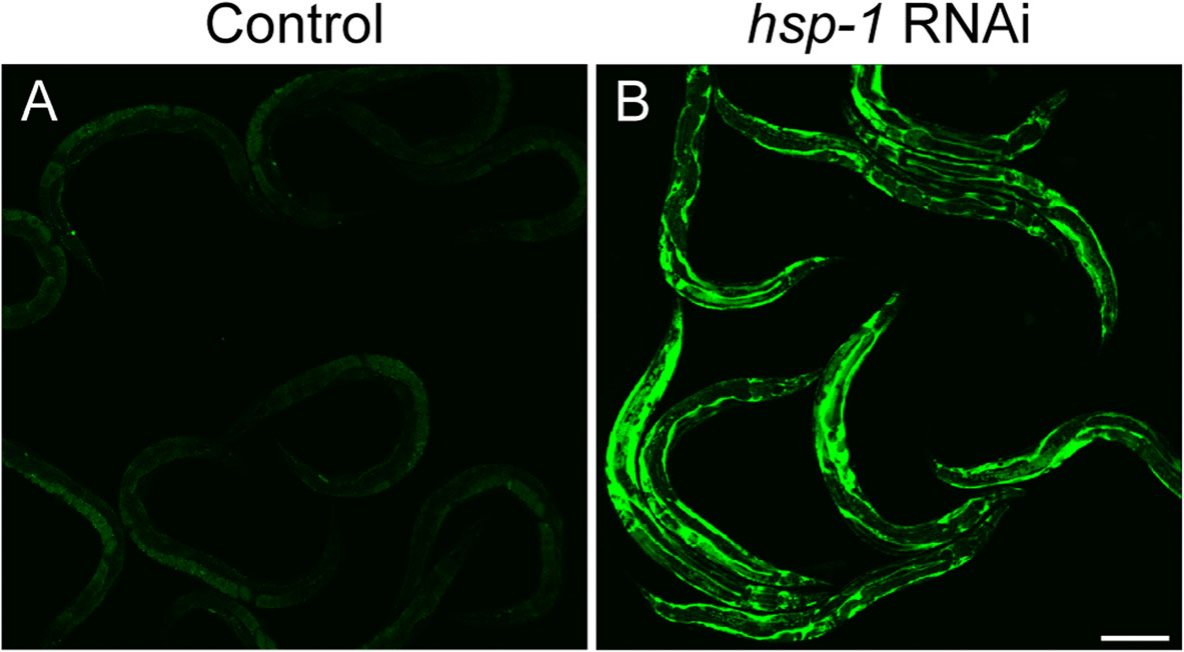
*hsp-1* knockdown inhibits yolk endocytosis. *hsp-1* knockdown causes yolk to accumulate in the pseudocoelomic space, as shown in YP170::GFP (RT130) worms that were raised to the L4 stage on OP50 and then exposed to either **a** control (L4440, empty vector) or **b** *hsp-1* RNAi for 48 h at 20 °C. Shown are representative images from three independent trials with *n* ≥ 10 worms. Scale bar: 150 μm

If chaperone titration disrupts endocytosis, then reducing or increasing chaperone levels would exacerbate or suppress endocytic defects, respectively. To test this, we first inhibited the predominant cellular HSP70 chaperone, *hsp-1*, using RNAi knockdown. At the control temperature of 20 °C, knockdown of *hsp-1* in the YP170::GFP reporter resulted in pseudocoelomic YP accumulation that mirrored chronic HS (Fig. 4). Therefore, depletion of HSP70 disrupts YP trafficking, consistent with previous reports demonstrating the role of HSP70 in endocytosis in other systems [13–15].

**Fig. 5:**
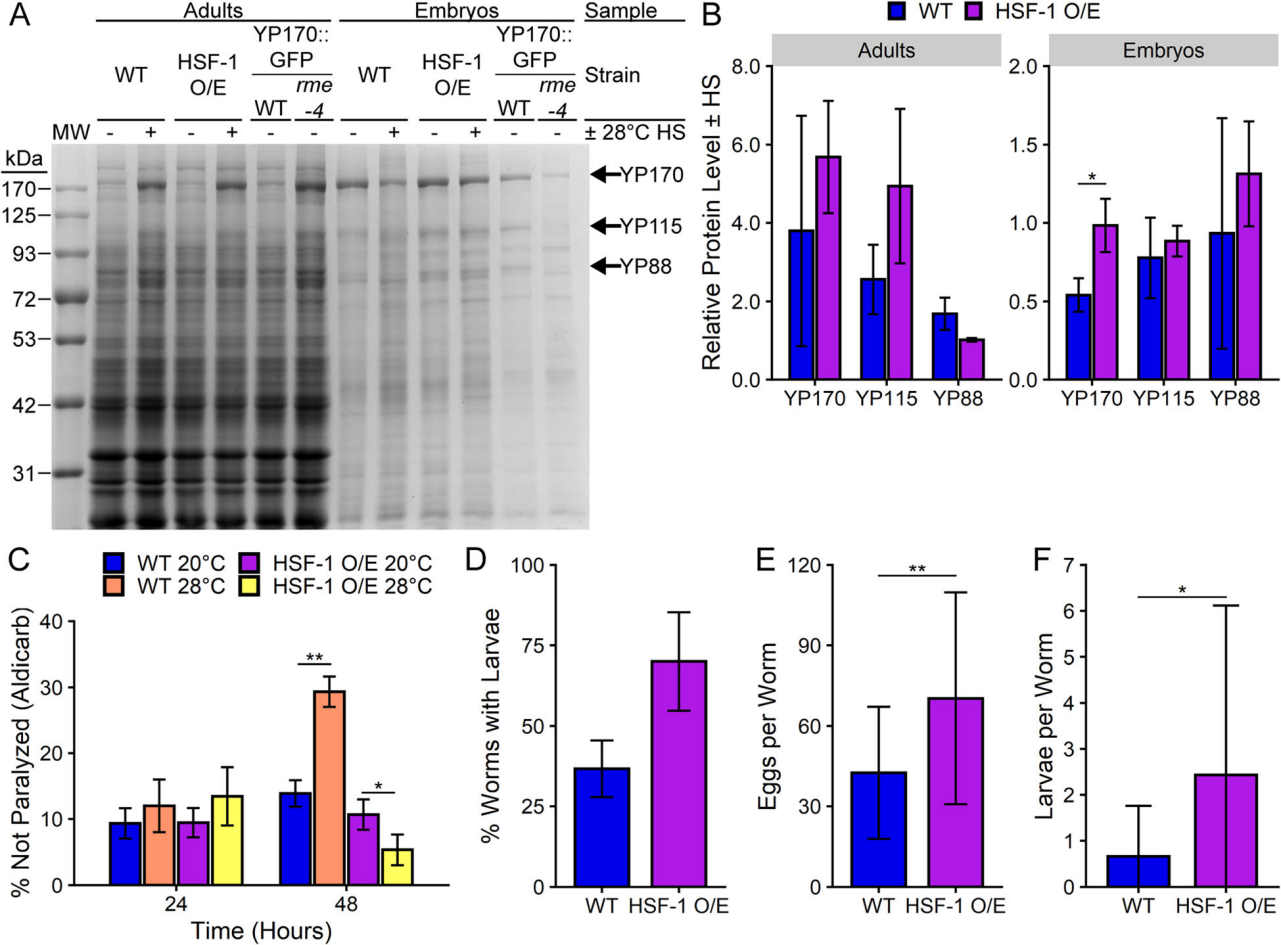
HSF-1 overexpression partially rescues yolk endocytosis, neuronal signaling, and recovery of offspring following chronic HS. **a**, **b** Coomassie-stained SDS-PAGE reveals that exposing adults to 24-h 28 °C HS results in a loss of YP170 in WT (N2) embryos that is rescued in HSF-1 overexpression (HSF-1 O/E; EQ140) embryos. **a** Representative image from one of three independent trials. Arrows indicate YP170, YP115, and YP88 bands. The *rme-4(b1001)* mutant YP170::GFP strain was included for YP band identification. MW: molecular weight marker; WT: wild-type. **b** Quantification of **a** shows the relative change in each YP level upon HS. Data represent the mean ± SD of *n* = 3 independent trials. **c** HSF-1 O/E worms show restored sensitivity to aldicarb compared to WT worms that show resistance to aldicarb following exposure to 48-h 28 °C HS. Neither strain shows significant changes in drug response after 24-h 28 °C HS compared to 20 °C controls. Data represent the mean ± SD of *n* = 3 independent trials. **d**–**f** HSF-1 O/E worms show improved reproductive function compared to WT during a 20 °C recovery period after a 24-h 28 °C HS, as shown by the percent of adults that recovered viable offspring (**d**) and the average number of eggs laid (**e**) and viable offspring produced (**f**) by each worm. Data in **d** represent the mean ± SD of *n* = 3 independent trials. Data in **e** and **f** represent the mean ± SD of *n* = 30 worms collected across three trials. * *p* < 0.05, ** *p* < 0.01 (Student’s *t* test)

Next, we tested the effects of increased HSP levels by using worms overexpressing the transcription factor heat shock factor 1 (HSF-1) [56]. To determine whether YP levels in the embryos were affected by chaperone levels, we collected equal numbers of eggs from wild-type and HSF-1 overexpression adults with and without HS. Consistent with a defect in endocytosis, isolated wild-type embryos contained lower levels of YPs per egg upon chronic stress (Fig. 5a). Quantitation of this effect showed that wild-type embryos had a 50% reduction in YP170 uptake capacity during HS (Fig. 5b). Remarkably, HSF-1 overexpression effectively restored embryonic YP accumulation, with these embryos maintaining ~ 98% of their YP170 uptake capacity. YP115 and YP88 also showed similar trends of rescue. However, HSF-1 overexpression was not sufficient to rescue YP accumulation inside adults during stress (Fig. 5a). Together, these results indicate that overexpression of chaperones is sufficient to rescue the YP endocytosis defect in oocytes during chronic stress.

We next tested whether chaperone titration represents a mechanism that generally affects cellular endocytosis by examining the effects of HSF-1 overexpression on neuronal signaling. HSF-1 overexpression reversed the aldicarb resistance induced by 48 h at 28 °C (Fig. 5c). Therefore, overexpression of HSF-1 can rescue multiple endocytosis defects and is not specific to YPs. The effects of HSF-1 overexpression are presumably through upregulation of chaperones; however, this data does not exclude other pathways regulated by HSF-1. Interestingly, no significant differences in aldicarb sensitivity were observed in wild-type or HSF-1 overexpression worms after 24-h 28 °C HS. This indicates that neuronal signaling is more resistant to stress-induced changes than the other pathways that showed endocytic defects at this timepoint.

Given that HSF-1 overexpression restored the endocytic defect in oocytes and the neuronal phenotype in adults, we next examined whether HSF-1 overexpression could protect reproductive output during chronic HS. Although HSF-1 overexpression was not sufficient to restore egg laying during continuous 28 °C HS, it did enhance recovery of viable offspring after a 24-h exposure to 28 °C HS (Fig. 5d). In these conditions, 100% of adults from both the wild-type and HSF-1 overexpression strains laid eggs during a 20 °C recovery period, but the HSF-1 overexpression worms laid significantly more eggs (Fig. 5e) and produced more larvae (Fig. 5f). Notably, only HSF-1 overexpression worms were capable of producing viable offspring in the first 24 h of recovery, whereas wild-type worms did not produce viable offspring until the second day of recovery (see Additional file 2: Fig. S7). These data suggest that increased chaperone levels can provide a stronger recovery of viable offspring following a period of chronic HS. Together, the recovery of YP endocytosis, neuronal signaling, and viable offspring indicate that a persistent disruption of proteostasis during chronic stress titrates chaperones and that this is a common molecular mechanism that contributes to multiple physiological processes.

## Discussion

**Fig. 6.**
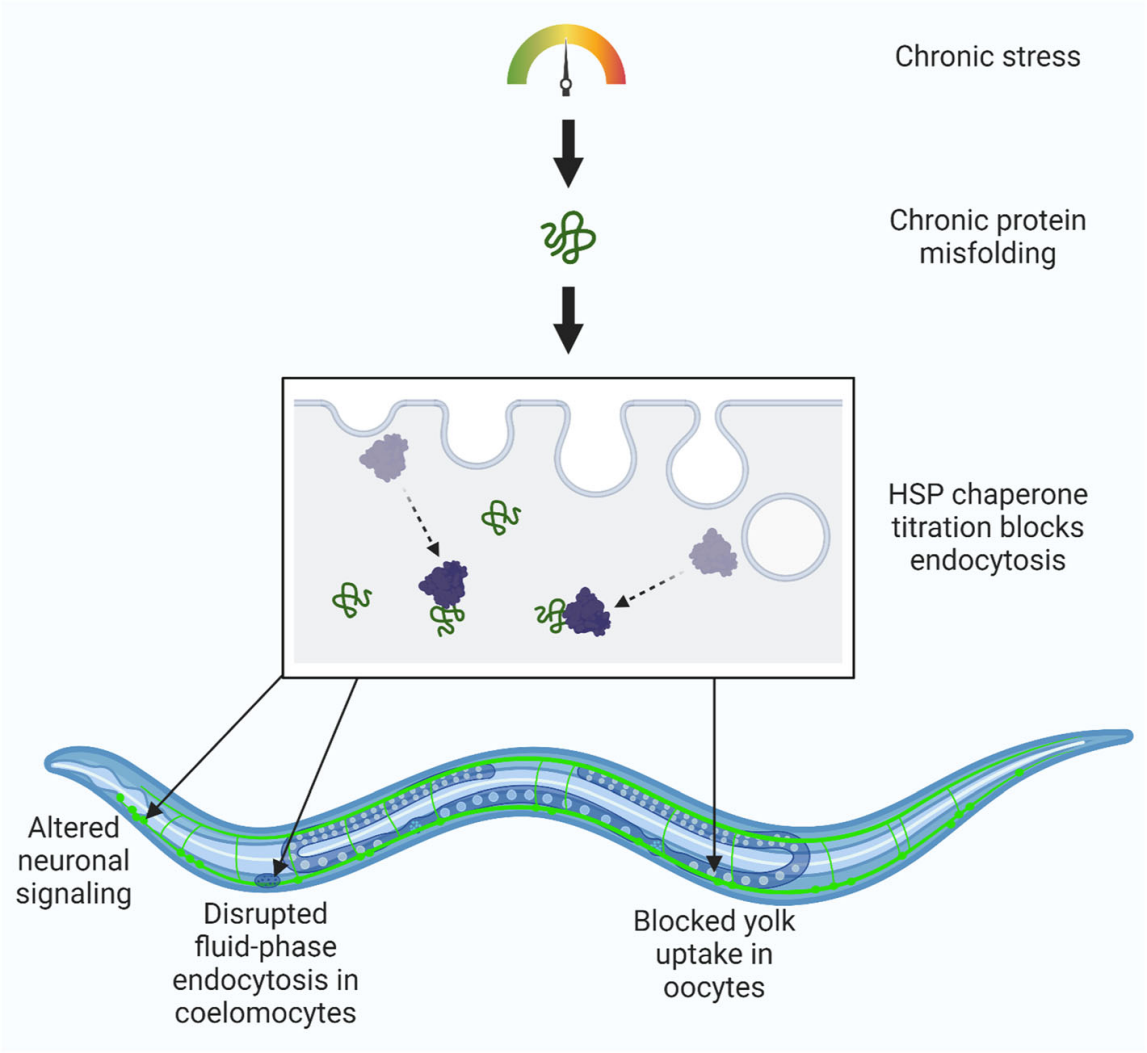
Chaperone titration and disrupted endocytosis link chronic heat stress with organismal phenotypes. Chronic protein-folding stress causes a titration of chaperones and subsequent disruption in endocytosis across cell types, ultimately leading to diverse physiological defects. Here, we provide evidence of this process in endogenous yolk levels and a fluorescent yolk reporter for embryos, a GFP reporter for coelomocytes, and functional assays for neurons coupled with HSF-1-mediated rescue of endogenous yolk uptake and neuronal signaling

We have identified chaperone titration as a molecular mechanism that contributes to a range of cellular and organismal phenotypes during exposure to chronic temperature stress (28 °C) (Fig. 6). The organismal phenotypes include cessation of egg laying and altered neuronal signaling. The cellular phenotypes include inhibition of YP uptake by oocytes and fluid-phase endocytosis by coelomocytes. A common feature of these phenotypes is their dependence on endocytosis. Our data indicate that chronic but mild temperature stress titrates chaperones away from their constitutive roles in endocytosis. Validating this model, induction of chaperones through HSF-1 overexpression partially rescues these phenotypes. Together, this work uncovers a unifying mechanism that connects molecular, cellular, tissue-specific, and organismal responses to chronic temperature stress.

Recent experiments have suggested that inhibition of endocytosis via chaperone titration plays an important role in multiple neurodegenerative diseases [57]. It was shown that ectopic expression of disease-associated, aggregation-prone proteins in cultured cells leads to endocytic disruptions that can be rescued by chaperone overexpression. Here, we demonstrate that a general temperature stress can have the same effects on chaperones and endocytosis as those seen with specific aggregation-prone proteins. Therefore, our work extends the model of chaperone titration-induced endocytic defects to an intact organism. Furthermore, the discovery that chaperone titration is a shared mechanism may help to explain why neurodegenerative diseases have such a large environmental component.

Our investigation has also uncovered critical spatiotemporal aspects of the responses to chronic temperature stress. A uniform, mild stress affects several processes central to organismal health, but at different timescales and with varying degrees of severity. The first observed phenotype in response to HS is a disruption of yolk trafficking into embryos seen in the first 6 h of 28 °C HS. By 24 h of HS, egg laying is terminated and fluid-phase endocytosis in coelomocytes is compromised. After 48 h of HS, neuronal signaling in response to aldicarb is altered and motility is slightly decreased. Thus, different tissues display unique temporal sensitivities to a stress applied uniformly to the organism. Distinct tissue-specific responses have also been observed through genetic disruption of protein folding, as tissues maintain unique balances between components of the proteostasis network and their tissue-specific substrates [58]. In addition, protein-misfolding mutations in a broadly expressed protein can elicit a “bystander” effect whereby the folding and trafficking of a specific but unrelated protein can be disrupted, possibly through titration of a selective, shared chaperone [59]. This effect was shown to produce cell-specific phenotypes despite the ubiquitous expression of the mutated protein. Recent work has also suggested cell-specific requirements for broad-specificity chaperones [60]. Therefore, the chronic stress-mediated phenotypes uncovered here contribute to the growing recognition of a range of cellular proteostasis capacities existing within an organism.

The earliest observed chronic stress phenotype, YP mislocalization at 6 h, precedes the reproductive defect. However, the reproductive defect is not likely to be caused by YP mislocalization alone, since YPs are not required for oogenesis, fertilization, or egg laying. Several mutations that block YP production do not affect egg laying, and mutations in the yolk receptor (RME-2) do not prevent production of viable offspring [47, 49, 61]. The observation that HSF-1 overexpression partially rescues reproduction after 24 h of stress indicates that chaperone titration contributes to the reproductive defect independently of yolk trafficking. However, since the rescue is not complete, there must be additional mechanisms behind this reproductive cessation. As temperature stress also disrupts embryonic development [62], these other mechanisms could include signaling pathways that coordinate development of the embryos with egg-laying behavior in the adult.

Notably, the repression of *vit* genes occurs after the YP trafficking defect. However, there is no visible accumulation of YP in the intestine, indicating that this effect is cell non-autonomous. Together, these data suggest a signaling pathway between the site of YP accumulation in the pseudocoelom and the site of synthesis in the intestine.

The framework we have established here provides a solid foundation for further investigations into the molecular and organismal responses to elevated temperature. The impact of this research is not limited to reproduction, as we have uncovered a wealth of gene expression changes induced by chronic stress. These gene expression changes are strongly enriched for specific categories, such as collagen genes, that are associated with specific organismal biology, such as cuticle structure. Our analysis connecting organismal responses to temperature with tissue-specific, cellular, and molecular pathways has wide-ranging implications for agricultural, ecological, and evolutionary studies. Furthermore, understanding the effects of extended but mild temperature stress grows increasingly critical in the face of elevated temperatures due to climate change.

## Conclusions

We have established *C. elegans* as a model organism for the systematic analysis of the effects of chronic stress. We have identified chaperone titration as a molecular mechanism that contributes to defects in protein trafficking, disruption of neuronal signaling, and cessation of reproduction during exposure to extended temperature stress.

## Methods

### Worm strains and maintenance

The following *Caenorhabditis elegans* strains were used in this study: Bristol strain N2, RT130(pwIs23[YP170::GFP]), RT362(*rme-4(b1001);*pwIs23[YP170::GFP]), GS1912(arIs37 [*myo-3*p::*ssGFP* + *dpy-20*(+)] I), and EQ140(iqIs37[pAH76(*hsf-1*p::*myc-hsf-1*)+pRF4(*rol-6*p::*rol-6*(*su1006*)]). Unless otherwise stated, worms were maintained at 20 °C. Worm populations were synchronized for experiments by allowing day 2 adult hermaphrodites to lay eggs for 1–1.5 h. The synchronized egg populations were then raised at 20 °C until day one of adulthood, after the onset of egg laying (~ 72 h post-synchronization for the N2 strain). For all heat stress (HS) experiments, plates of day 1 adults were transferred to a 28 °C dry incubator (HS worms) for the specified time course.

### Egg-counting experiments

Synchronized wild-type (N2) worms at the late L4/young adult (YA) stage (~ 58 h after synchronization) were singled onto 35 mm NGM plates seeded with 100 μL OP50 and kept at 20 °C for 12 h to reach egg-laying adulthood. The individuals were then transferred to fresh 35 mm plates and shifted to 28 °C for HS or maintained at 20 °C for control conditions (this represents the 0-h timepoint) and the eggs laid on the first plate were counted. Worms were transferred to fresh, pre-warmed plates every 12 h with minimal exposure (< 5 min) to ambient temperatures.

### Motility assays

Thrashing was scored at room temperature (RT) in a 10-μL drop of M9 on NGM agar. Individual worms were placed into the droplet and allowed to acclimate for 30 s, then each body bend was counted for 30 s.

### RNA-sequencing

Synchronized day 1 adult wild-type (N2) worms were either collected immediately (0-h 20 °C control), or shifted to 28 °C HS for 1, 24, or 48 h before collection. RNA was collected as described previously [63]. Library preparation, sequencing, and initial data analyses were performed by Novogene. Total RNA was poly-A selected and then subjected to 150-bp paired-end Illumina HiSeq sequencing using three biological replicates for each condition. The raw reads were filtered to remove reads with adaptor contamination, reads consisting of > 10% uncertain nucleotides, and reads where > 50% of the bases were low quality (base quality < 20). The remaining clean reads (~ 97% for each sample) were mapped to the WBcel235 *C. elegans* reference genome. Approximately 92–93% of the clean reads were mapped per sample. Genes that had no mapped reads for control or HS conditions were excluded from the differential expression analysis. Differentially expressed genes were identified using DESeq (v.1.18.0) by normalizing reads based on the negative binomial distribution method and comparing each HS timepoint to the 0-h control.

### Fluorescence microscopy

Worms were anesthetized in 0.5–1.0 mM levamisole on a 3% agarose pad for imaging, as described previously [63]. Images were analyzed using EZ-C1 software, Gold Version 3.90 build 869 (Nikon Corporation, Tokyo, Japan). The range and saturation levels of each image were normalized across conditions at each timepoint to allow for direct comparisons. Each trial scored *n* ≥ 10 worms per condition.

### SDS-PAGE and Coomassie staining

For adult protein lysates, equal numbers of adults (50–60) from each condition were picked into microcentrifuge tubes containing 33 μL Laemmli sample buffer. Samples were then boiled for 10 min, centrifuged for 10 min at 14000 x RPM at RT, and the supernatants were transferred to fresh tubes. Equal volumes were loaded per lane.

For embryo lysates, ~ 100–200 adults from each condition were collected, and fresh bleach solution (20% sodium hypochlorite + 0.25 M NaOH in ultrapure H_2_O) was added to each tube to dissolve the adults. After ~ 8 min with gentle mixing (or until adults were dissolved), embryos were washed once with M9, resuspended in 15 μL M9, and then the number of eggs was counted using a dissecting scope. Samples were then mixed with 2x Laemmli sample buffer, boiled, and centrifuged before loading. Equal numbers of eggs (~ 800) were loaded per lane.

Lysates were loaded into a freshly prepared 8% SDS-PAGE. Gels were stained using Coomassie brilliant blue and imaged using a ChemiDoc™ XRS System (Bio-Rad Laboratories Inc., Hercules, CA).

### Yolk protein quantification

YP bands were identified based on comparisons to literature and validated by comparing banding patterns with RME mutants known to accumulate YPs [44–46]. Using ImageLab 6.0.0 (Bio-Rad), each gel was quantified by normalizing background levels and then calculating each adjusted YP170, YP115, and YP88 band volume relative to the total lane volume for that sample. The relative accumulation or loss of each YP was calculated by dividing the adjusted YP volume in the HS lane by the adjusted YP volume in the control lane for that timepoint.

### RNAi experiments

RNA interference (RNAi) was performed against the indicated genes, with L4440 as the vector control, using feeding RNAi in *E. coli* HT115(DE3) [64]. RNAi clones were sequence-verified before use. RNAi against *ceh-60* was initiated from egg-lay synchronization. RNAi against *hsp-1* was initiated in L4 worms to avoid developmental defects and was carried out for 48 h at 20 °C before imaging.

### Neuronal transmission assays

Paralysis plates were made using NGM containing final concentrations of 1 mM aldicarb (Adipogen Corporation, San Diego, CA) or 0.5 mM levamisole (Acros Organics, Thermo Scientific, Waltham, MA). For each paralysis assay, drug plates were freshly seeded with OP50 and dried at RT for 24 h. Then, 25 adult worms that had been exposed to 20 °C (control) or 28 °C (HS) for 24 or 48 h (as described for HS conditions) were picked to each drug plate and placed in a 20 °C incubator. Levamisole sensitivity was scored 2 h after plating and aldicarb sensitivity was scored 8 h after plating. Worms were scored as paralyzed if they did not move after being gently prodded 3x on the head and tail with a platinum wire pick. Worms that ruptured or crawled up the sides of the drug plate were censored. The percentage of worms not paralyzed in each trial was calculated from the total number of non-censored worms for that plate.

### Reproduction recovery experiments

Synchronized day 1 adult wild-type (N2) or HSF-1 overexpression (EQ140) worms were singled onto 35 mm OP50 plates, shifted to 28 °C HS for 24 h, and then shifted back down to 20 °C for recovery. During the recovery period of up to 7 days, adults were transferred to fresh OP50 plates daily and the eggs on each plate were counted. The same plates were scored again the following day for appearance of larvae (viable offspring).

### Statistics and figures

Graphs were prepared with accompanying statistics using R version 4.0.4 (R Foundation for Statistical Computing, Vienna, Austria). A *p* value < 0.05 was considered statistically significant. Model figures (Figs. 3e and 6) were created with BioRender.com.

## Supplementary Information

**Additional file 1. **DEGs. Worksheet containing the DESeq output file containing statistically differentially expressed genes from each timepoint with read counts, log_2_ fold-change, and *p*-values.

**Additional file 2: Figure S1-S7.** File containing all supplemental figures. **FigS1.** Venn diagram comparing Acute (1-h), Mid (24-h), and Chronic (48-h) 28 °C HS differentially expressed genes. **FigS2.** Venn diagram comparing Chronic (48-h) 28 °C HS and Classical Acute HSR differentially expressed genes. **FigS3.** YP170::GFP reporter ±24-h 28 °C HS at 20x and 40x magnification. **FigS4**: GO term enrichment analyses highlight dynamic stress-induced transcriptomes. **FigS5.** Oogenesis-enriched genes show temporal response to stress. **FigS6.** Knockdown of YP with *ceh-60* RNAi does not restore coelomocyte endocytosis during 24-h 28 °C HS. **FigS7.** HSF-1 overexpression provides faster recovery of offspring after 24 h of 28 °C HS.

**Additional file 3: Gene lists.** Worksheet with separate tabs for: **FigS1**, all differentially expressed genes shared between Acute (1-h), Mid (24-h), and Chronic (48-h) 28 °C HS; **FigS2**, all differentially expressed genes shared between Chronic (48-h) 28 °C HS (this work) and Classical Acute HSR genes (33 °C HS for 30 min, from Brunquell et al. [35]); **FigS3**, GO analysis results including all significantly enriched GO terms and the up- and down-regulated genes from each term for each 28 °C HS timepoint (listed by WormBase ID).

## Data Availability

The datasets supporting the conclusions of this article are included within the article and its additional files. Complete sequence reads are available in the Sequence Read Archive (SRA) at NCBI under project number PRJNA705210 [65].

## Abbreviations

HS: Heat stress
HSF: Heat shock factor
HSP: Heat shock protein
HSR: Heat shock response
RME: Receptor-mediated endocytosis
ssGFP: Signal-sequence green fluorescent protein
YP: Yolk protein
