# Supplementary material for: Chronic temperature stress inhibits reproduction and disrupts endocytosis via chaperone titration in Caenorhabditis elegans
Source: BMC Biol. 2021 Apr 15;19:75. doi: 10.1186/s12915-021-01008-1 (PMC8051109; doi:10.1186/s12915-021-01008-1)
Supplement: Supplementary file 2 — Additional file 2: Figure S1-S7. File containing all supplemental figures. FigS1. Venn diagram comparing Acute (1-h), Mid (24-h), and Chronic (48-h) 28 °C HS differentially expressed genes. FigS2. Venn diagram comparing Chronic (48-h) 28 °C HS and Classical Acute HSR differentially expressed genes. FigS3. YP170::GFP reporter ±24-h 28 °C HS at 20x and 40x magnification. FigS4: GO term enrichment analyses highlight dynamic stress-induced transcriptomes. FigS5. Oogenesis-enriched genes show temporal response to stress. FigS6. Knockdown of YP with ceh-60 RNAi does not restore coelomocyte endocytosis during 24-h 28 °C HS. FigS7. HSF-1 overexpression provides faster recovery of offspring after 24 h of 28 °C HS. [file 12915_2021_1008_MOESM2_ESM.pdf]

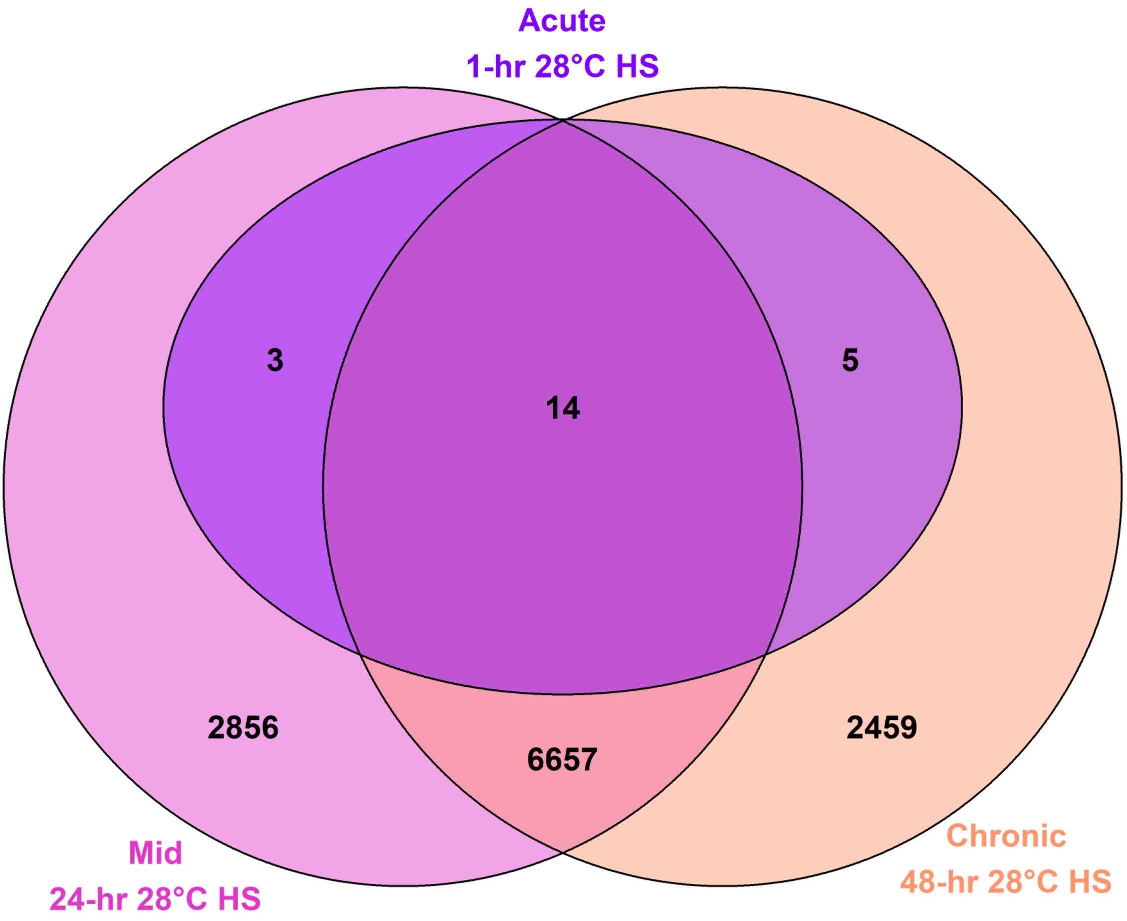

Figure S1: Venn diagram comparing Acute (1-hr), Mid (24-hr), and Chronic (48-hr) 28°C HS differentially expressed genes. All differentially expressed genes compared across the chronic 28°C HS time course. Lists of genes in each overlapping (shared) region can be found in AdditionalFile3_GeneLists.xlsx.

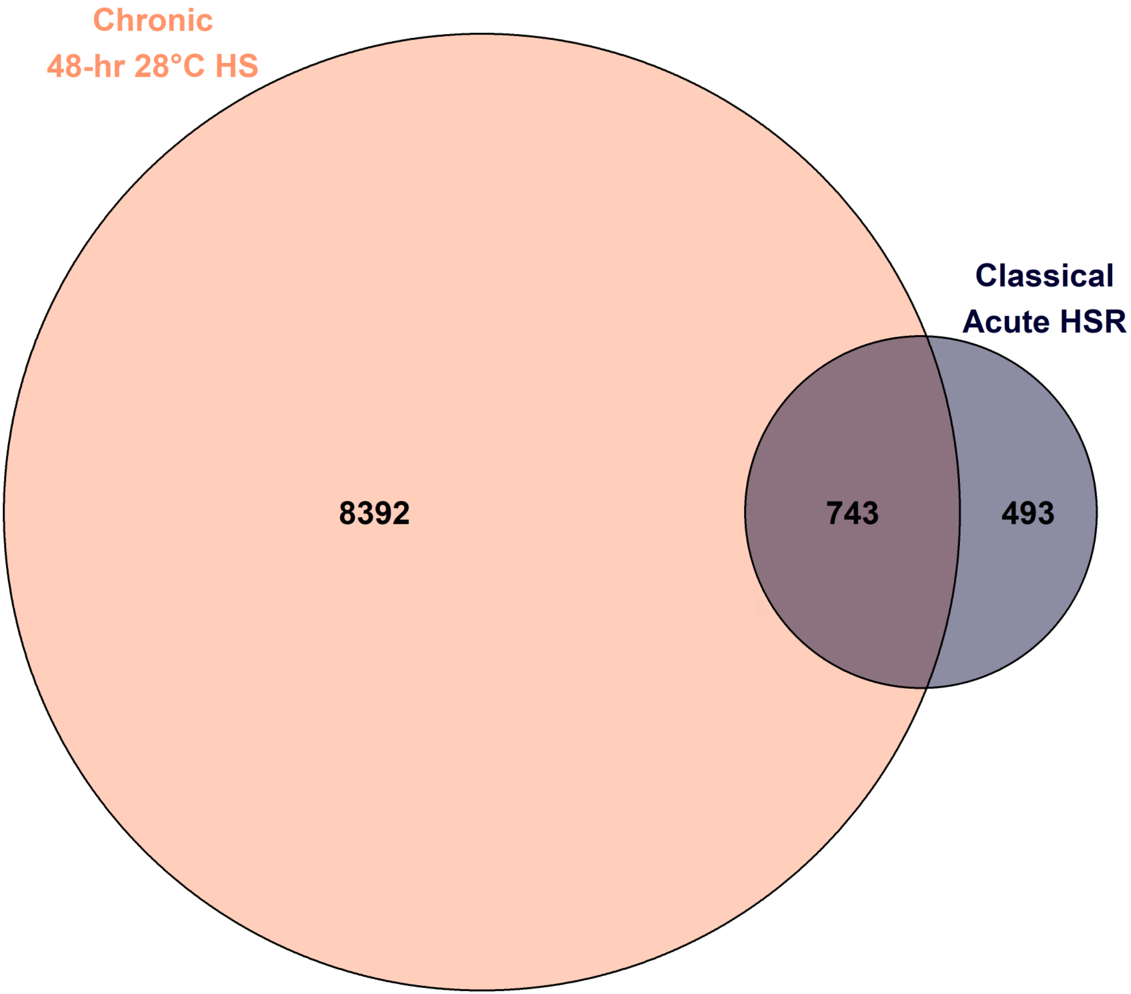

Figure S2: Venn diagram comparing Chronic (48-hr) 28°C HS and Classical Acute HSR differentially expressed genes. All differentially expressed genes from chronic, 48-hour 28°C HS (this work) compared with genes differentially expressed by a classical acute HS (30-minute 33°C HS) from Brunquell et al. (ref [35]). Lists of all genes and of the overlapping (shared) genes can be found in AdditionalFile3_GeneLists.xlsx.

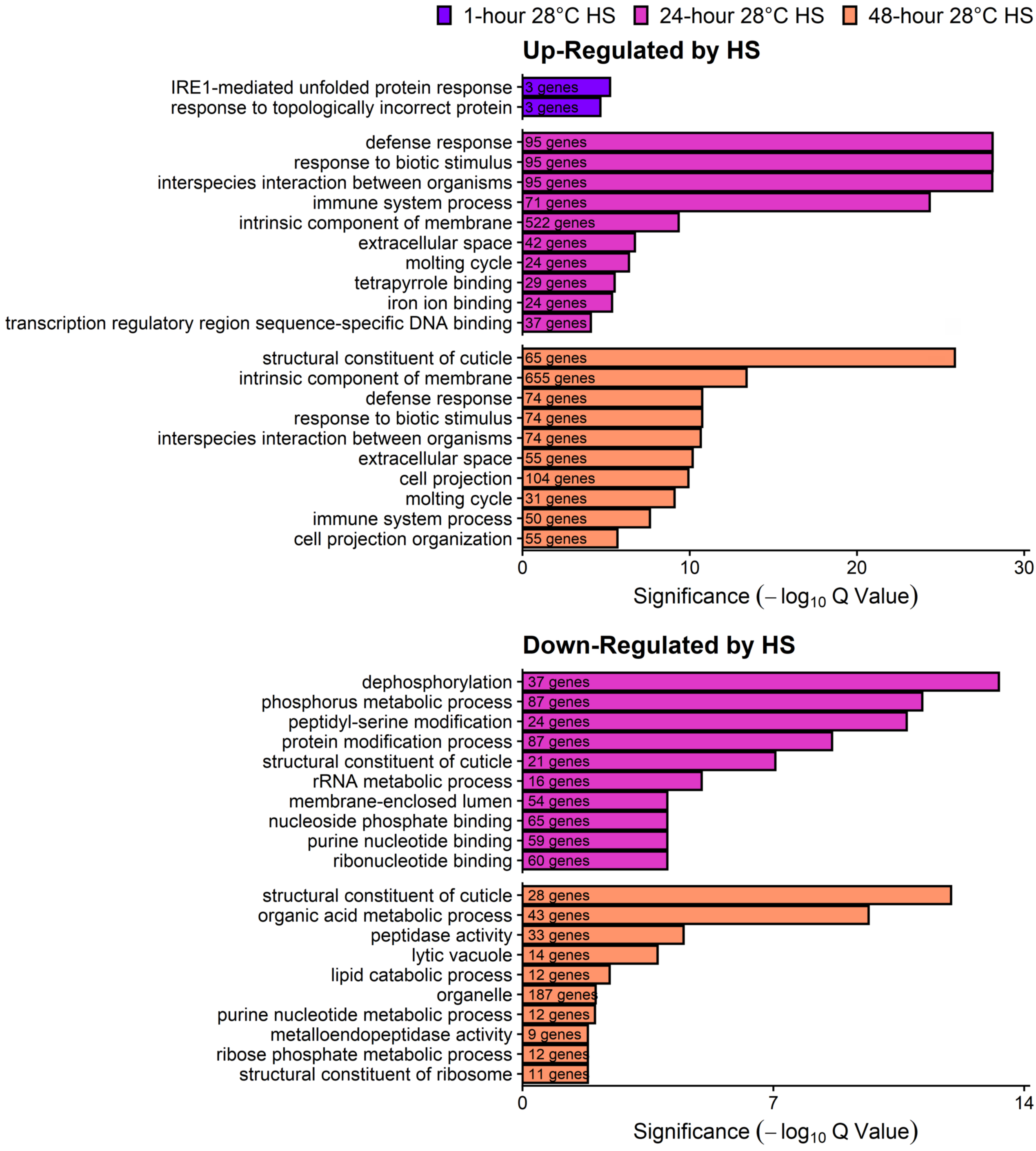

Figure S3: GO term enrichment analyses highlight dynamic stress-induced transcriptomes. GO term enrichment was analyzed using RNA-seq data collected from WT (N2) hermaphrodites following exposure to 1-hour, 24-hour, or 48-hour 28°C HS initiated on day 1 of adulthood. The top 10 enriched GO terms among the genes that were up- or down-regulated ≥ 4-fold relative to 0-hour (20°C) controls are shown as the significance of the GO enrichment (-log10 of the Q value). The number of up- or down-regulated genes in each term are indicated in each bar. See AdditionalFile3_GeneLists.xlsx.

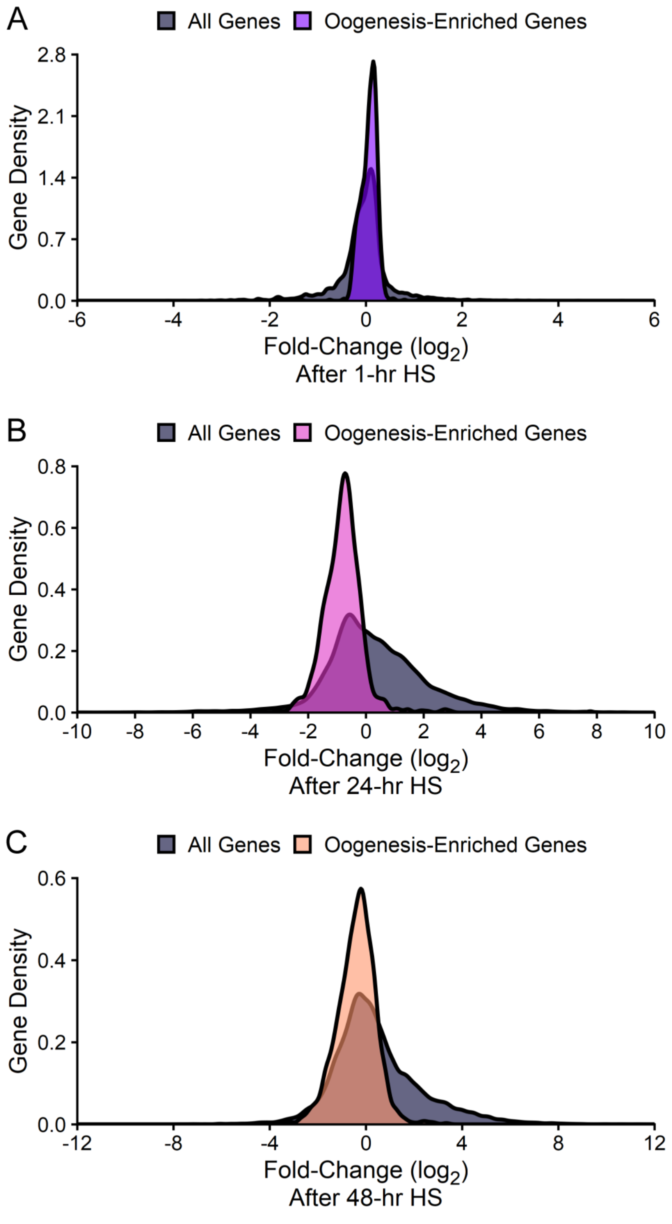

Figure S4: Oogenesis-enriched genes show temporal response to stress. Analysis of oogenesis-enriched genes reveals time-dependent responses to 28°C HS in adult WT (N2) worms. Curves represent the densities of all genes (gray) or oogenesis-enriched genes (color) at each expression fold-change (log2) value relative to the 0-hour (20°C) controls following 1-hour (A), 24-hour (B), or 48-hour (C) 28°C HS as determined by RNA-seq analysis.

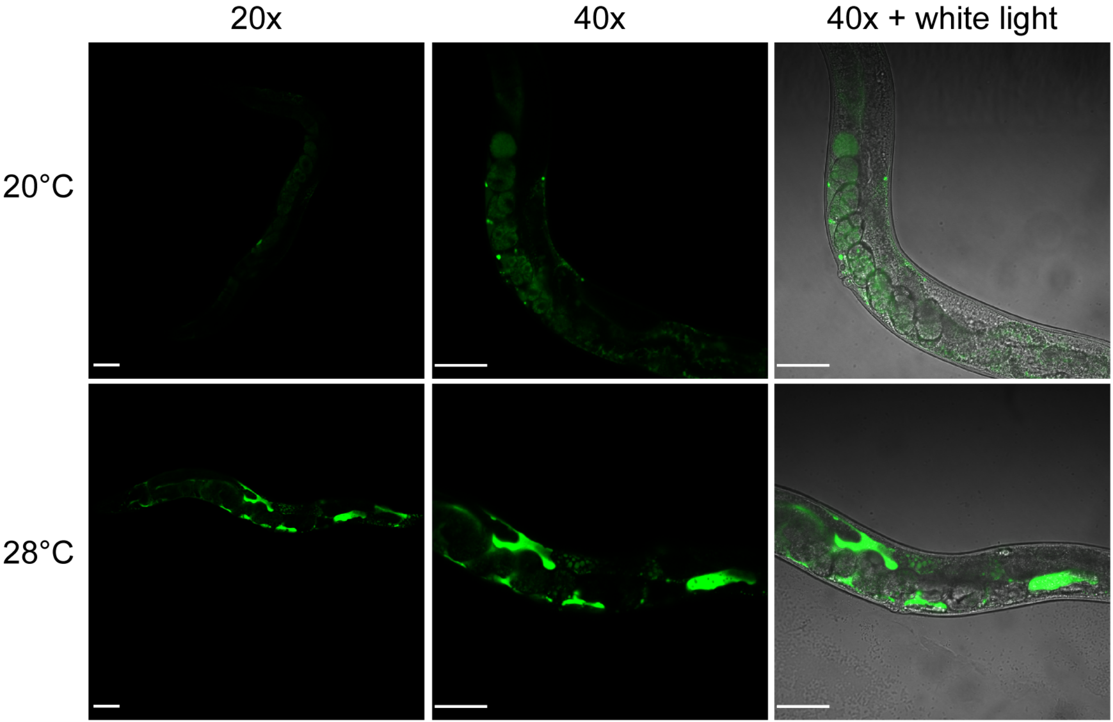

Figure S5: YP170::GFP ± 24-hour 28°C HS at 20x and 40x magnification. Day 1 adult YP170::GFP (RT130) hermaphrodites were exposed to 24 hours of 20°C (control) or 28°C HS before imaging. Scale bar: 50 µm.

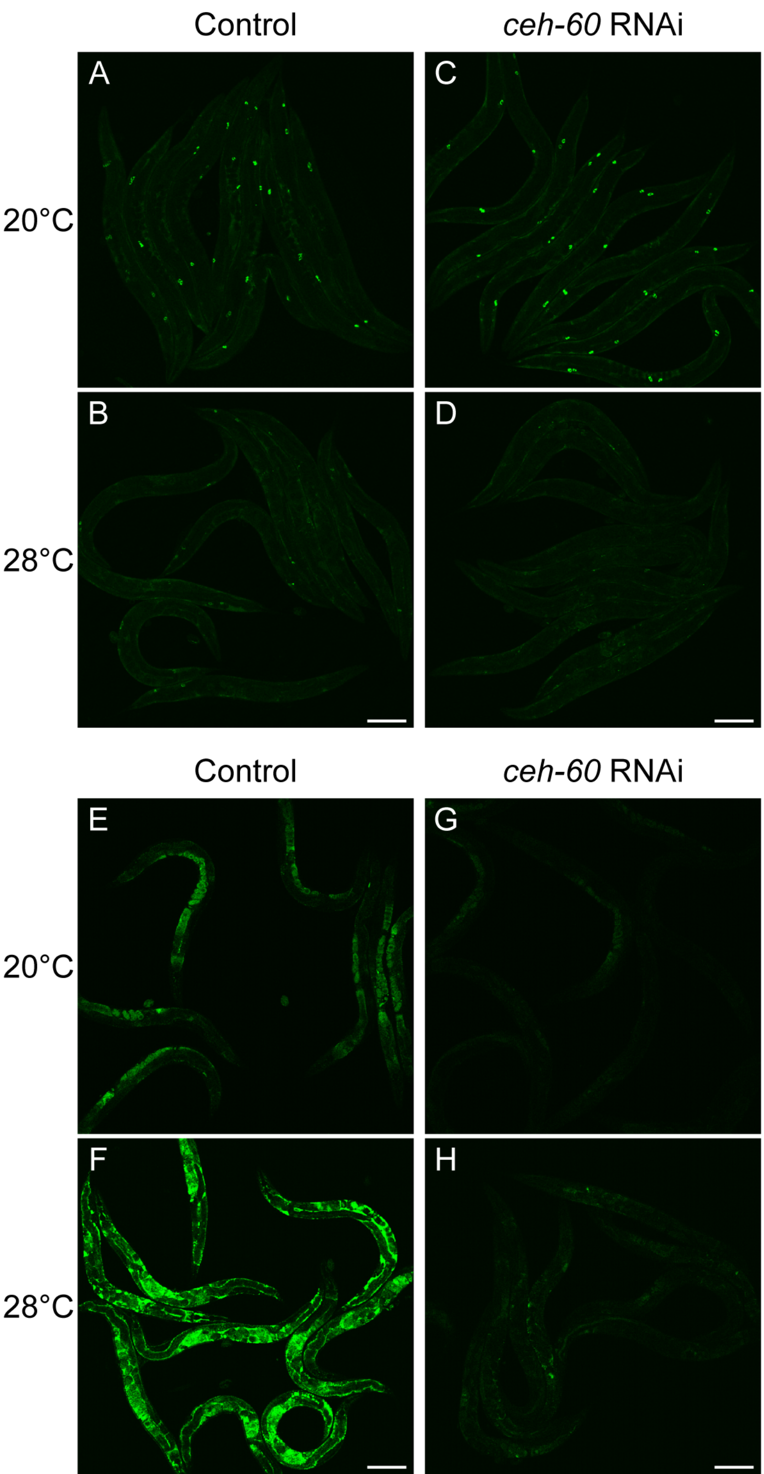

Figure S6: Knockdown of YP with ceh-60 RNAi does not restore coelomocyte endocytosis during 24-hour 28°C HS. The effects of yolk knockdown during chronic stress were assessed using hermaphrodites that were raised from egg to day 1 adults on either control (L4440, empty vector) or ceh-60 RNAi before being exposed to a 24-hour 28°C HS. A-D) Yolk knockdown has no effect on coelomocyte endocytosis at control (20°C) or HS temperatures, as observed in myo-3p::ss-GFP (GS1912) worms. E-H) Yolk knockdown efficiency is demonstrated in YP170::GFP (RT130) worms. Shown are representative images from three independent trials with n ≥ 10 worms. Scale bar: 150 μm.

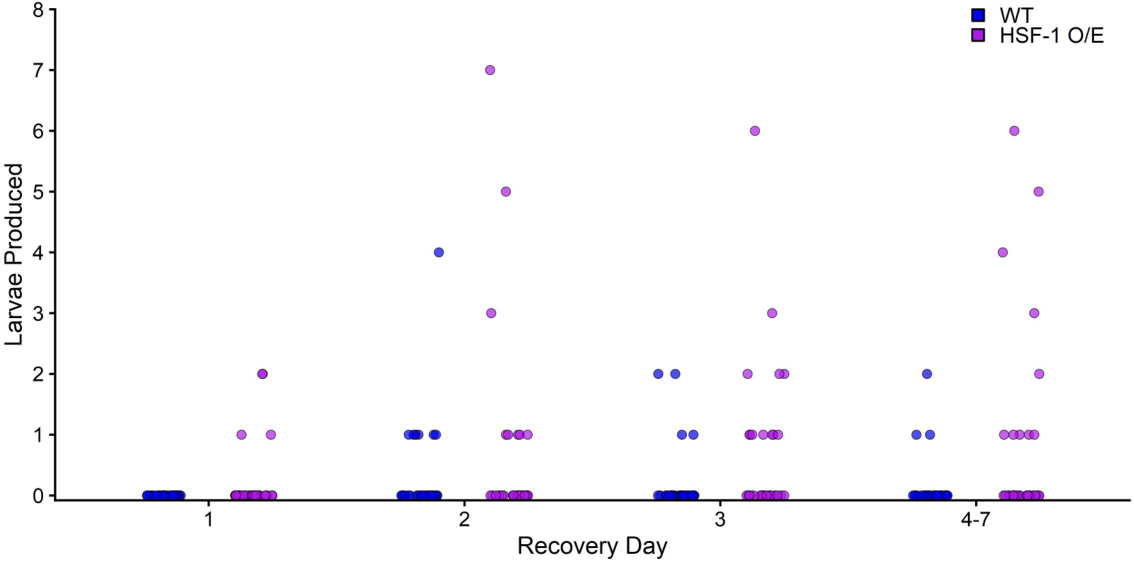

Figure S7: HSF-1 overexpression provides faster recovery of offspring after 24 hours of 28°C HS. HSF-1 overexpression (HSF-1 O/E; EQ140) worms show faster recovery of viable offspring compared to WT (N2) during a 20°C recovery period after a 24-hour 28°C HS, as shown by the number of larvae produced daily by each worm. Data represent n = 30 worms collected across three trials, where the larval counts for individual worms are pooled from recovery days 4-7.
